# ACQUIRED: An Innovative Asynchronous Modality to Increase Quality Teacher-Learner Dialogue and Overcome Classroom Barriers in Basic Science Medical Education

**DOI:** 10.1007/s40670-024-02248-w

**Published:** 2025-02-06

**Authors:** Brianne E. Lewis, Stefanie M. Attardi, Kara E. Sawarynski

**Affiliations:** 1 Department of Foundational Sciences, Central Michigan University College of Medicine, Mount Pleasant, MI USA; 2 Department of Foundational Medical Studies, Oakland University William Beaumont School of Medicine, Rochester, MI USA

**Keywords:** Quality education, Medical education, Asynchronous, Online, Instructional design

## Abstract

Learners are faced with psychological and temporal classroom barriers that may preclude participation and learning. We present an asynchronous instructional design modality aiming to address learning barriers, retain active learning, and foster quality teacher-learner interactions. We designed and evaluated individualized online lessons constructed with flexible navigation, short instructional videos, integrated formative assessment, and reflection questions with bidirectional teacher-learner communication. Narrative student comments (*n* = 3075, from 33 basic science sessions over three cohorts of 125 students) were extracted from faculty evaluations, and descriptive coding identified strengths and constructive criticisms. Data are presented as mean ± SD. A majority of comments were positive (76.6% ± 2.12%) with 43.5% ± 8.7% of these focused on the ACQUIRED modality. Within these responses, students most benefited from formative practice questions (18.6% ± 2.3%), the modular format (17.3% ± 2.7%), engagement with material (11.1% ± 0.9%), ease of asking questions (9.2% ± 2.4%), overall organization (8.0 ± 2.8%), and fast responsiveness from the teacher (7% ± 2.0). Fewer comments were constructive (15.0 ± 2.1%), with 51.5% ± 30.9% of these focused on the ACQUIRED modality. More formative questions (30.5% ± 21.8%) were described for improvement. Our data informed the design of the ACQUIRED model for asynchronous instruction, emphasizing **AC**cessible **QU**ality **I**nteractions and **RE**sponsive **D**esign. The model addresses learning and participation barriers and fosters meaningful, bidirectional teacher-learner interactions.

**Supplementary Information:**

The online version contains supplementary material available at 10.1007/s40670-024-02248-w.

## Introduction

The vast majority of health profession students in the USA are members of Generation Z (Gen Z) [1]. Born after 1994, their life experiences are unique from previous generations, which may impact how they prefer to participate in higher education [2–4]. Despite having a preference for active learning strategies, learners may hesitate to participate [2, 5, 6]. Further, Gen Z learners have described their real learning as taking place outside of the classroom setting, voicing a preference to have resources to use on their own time [6]. To date, Gen Z learners have spent more time with remote learning options which may impact readiness to participate in face-to-face learning modalities [7].

**Fig. 1:**
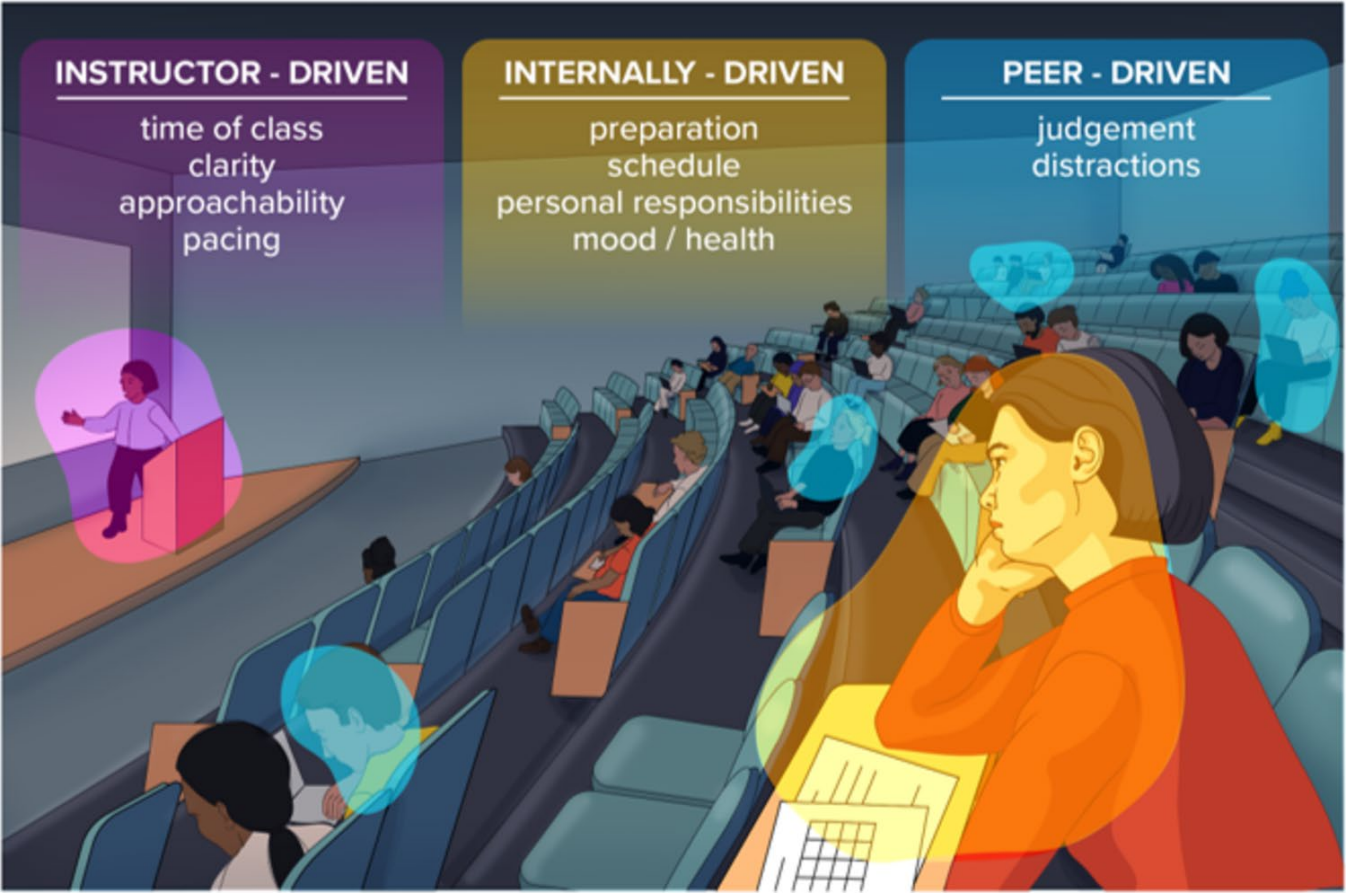
Intrinsic and Extrinsic barriers to student participation in the classroom. Several factors, whether instructor-, self-, or peer-driven can impact the learner’s ability or willingness to participate in the classroom

Barriers to participating in face-to-face learning modalities can be described by a combination of intrinsic and extrinsic influences which are exacerbated by Gen Z learner preferences [8, 9]. Extrinsic or intrinsic factors affecting learning in the classroom may arise from the instructor, self, or peers (Fig. 1). *Instructor-driven factors*, such as teacher preferences for providing instruction, may not align with learners’ preferences. For example, Gen Z learners have a strong preference for and reliance on technology with a sense of immediacy for communication, driven by their life experiences [9, 10]. This is in contrast to previous generations’ experiences where life was less technology-dependent, and classrooms were not heavily integrated with multiple technologies. In addition, the teacher’s attitude, approachability, and classroom management have been shown to impact learning or participation [9, 11]. *Peer-driven behaviors* and teacher behavior can impact participation if feelings of judgment or a threat to psychological safety are experienced by the learner [12, 13]. Gen Z learners, in particular, demand a safe space to engage in discussion and learning [2, 3]. Additionally, barriers to participation in a classroom may be *internally driven*. These include students’ attitudes and expectations, level of preparation, schedule and personal time management, competing priorities, and mood and health [9, 14–16]. Finally, Gen Z learners are more diverse than previous generations [2]. Amplified by holistic admissions, learners entering health professions education have varied levels of scientific readiness and experiences [17, 18], all of which need to be balanced in the classroom. Irrespective of the teaching modality, teachers should plan to reduce barriers to participation by tailoring instruction to meet the needs of the learners, while not compromising the desired learning outcomes. Health professions educators are challenged to meet the educational needs of diverse learners, especially when and helping to acclimate learners to the highly rigorous and rigid context of the first semester.

While there are many learning modalities, asynchronous learning could provide a potential solution to classroom barriers by optimizing learning without sacrificing the high standards and structure of medical education. Asynchronous learning can be defined as instruction that is available for learners to access, use, and participate in at a time and place of their choosing [19]. Naturally, this definition lends itself to an array of instructional designs which are all technically considered asynchronous. For example, at its simplest form, asynchronous learning may be a teacher posting a pre-recorded video analogous to an in-person didactic lecture. To date, studies evaluating the efficacy of asynchronous or e-learning are mixed, in part due to the varying types of instructional design which makes it difficult to compare across modalities [20–24]. Depending on the instructional design, one criticism of asynchronous learning is the inability for learners to interact with the teacher through questions [25]. Further, studies that examined asynchronous learning only during emergency remote teaching may need to be re-evaluated as health restrictions are no longer driving the choice of learning modality [26].

Moore’s transactional distance theory, originally proposed in the context of distance learning, can be applied broadly to all instructional methods and delivery formats [27]. Moore argues that transactional distance, the psychological space between teacher and learner in which there is potential for misunderstanding, can be reduced by maximizing “instructional dialogue” and minimizing “structure” [27]. These elements may be thought of as “responsive teaching” and “quality and accessible interactions” and can explain how teacher behavior drives student learning. A responsive teacher is flexible and adapts to the needs of the learner (minimizes structure). Quality and accessible interactions provide purposeful, bidirectional communication between teacher and learner (dialogue). However, fostering teacher-learner interaction has been a challenge to implement in asynchronous learning modalities [28]. A recent study demonstrated that, for asynchronous learning, teachers and learners both report a pre-recorded lecture being the most frequently used strategy, with fewer interactive opportunities utilized [29]. Furthermore, while feedback is possible through asynchronous instruction, it is not designed into the learning modality as frequently compared to synchronous delivery methods [29]. As such, the existing literature reveals a gap in strategies aimed at reducing barriers to participation and fostering rapid, bidirectional communication, particularly with Gen Z learners. Asynchronous learning that is intentionally designed, grounded in theory, and supported with established best practices, could be modernized to meet the needs of current Gen Z learners [27, 30].

The current study addresses this gap in the literature through the following objectives:

1. Design a modality for asynchronous teaching of basic sciences in undergraduate medical education that is focused on facilitating bidirectional communication and minimizing barriers to participation for Gen Z learners in their first semester.
2. Describe and quantify the benefits of the design, as expressed by learners.
3. Describe and quantify aspects of the design that can be improved, as expressed by learners.

Herein, we present a type of asynchronous design, Individualized Online Lessons, and report an analysis of data collected from end-of-course faculty teaching evaluations, over three classes of first-year medical students. This article will provide an overview of the modality and culminate in a transferrable model with practical tips to aid medical educators in implementing this asynchronous design.

## Methods/Materials

### Study Design

This study used a multi-methods approach to inquiry, where qualitative coding methods were undertaken to describe narrative data from students, and descriptive statistics were calculated to quantify the codes. Quantitative analysis was necessary to distill and interpret our large narrative dataset of over 1000 statements.

### Research Setting

This study took place at Oakland University William Beaumont School of Medicine (OUWB), an allopathic medical school with a class size of 125 per cohort. The medical program is 4 years long, with the majority of foundational biomedical sciences taught in the first 2 (preclinical, M1, and M2) years followed by 2 years of clinical rotations. The Individualized Online Lessons used in this study were offered during the fall semester (August through December) in the years 2020, 2021, and 2022. In 2020, during the COVID-19 pandemic, all preclinical lectures were delivered online due to the university’s physical distancing mandate. Since 2021, all preclinical courses have been delivered in a hybrid format, with some lectures in-person and others held remotely online. Each faculty member has the opportunity to intentionally choose the best modality for their learning objectives, resulting in a variety of delivery modalities used within a course. The current study describes and evaluates a novel method of asynchronous teaching basic medical sciences, using first year biochemistry, cell biology, and histology lectures as examples. Each of these disciplines was part of integrated foundational science courses and was interspersed with laboratories, case-based problem-solving sessions, team-based learning (TBL), patient panels, and synchronous lectures. Attendance was mandatory during instructional methods that relied on peer-to-peer teamwork and patients, while lecture attendance was generally optional, although the content was covered on assessments. Summative assessments in the form of midterm or final examinations were held approximately every 4 weeks, occurring within the respective preclinical course. The examinations were instructor-written, multiple choice assessments in the clinical vignette-style of the National Board for Medical Examiners [31].

### Pedagogical Innovation: Asynchronous Lessons

The content and activities for the Individualized Online Lessons in this study were delivered using Moodle Lessons. While Moodle lessons were used at this institution, most learning management systems have analogous capabilities. Moodle (moodle.org) is a free, open-source learning management system (LMS) designed for education. Moodle Lesson is a tool in the LMS that allows the educator to custom-build content pages with branching scenarios. Content pages can display text, embedded multimedia resources, hyperlinks, and assessment questions (multiple choice, matching, true/false, short answer, or essay). Using Individualized Online Lessons, the teacher can pre-program which pages are displayed based on input from the learner. The teacher can view a report for each learner, including how they progressed through the Individualized Online Lesson in their individual scores on closed-ended assessment questions. Aggregate assessment data from all learners can also be accessed by the educator.

**Fig. 2:**
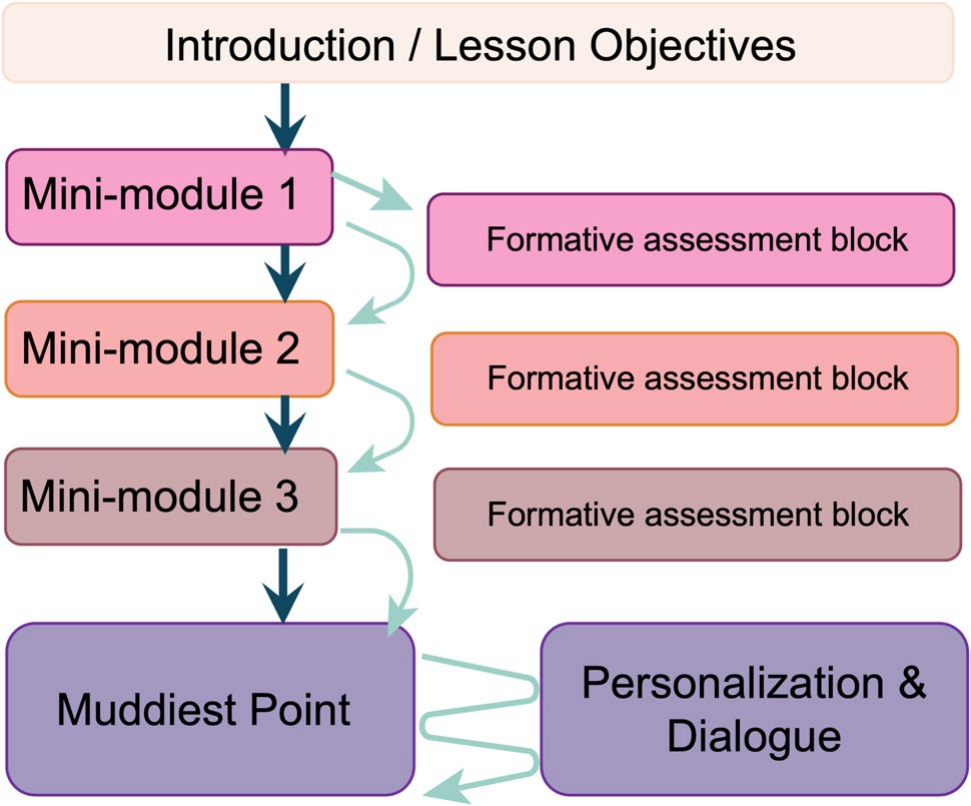
Individualized Online Lesson Structure and Workflow. Students first are presented with an introduction and description of learning objectives. Then they view several mini-modules containing short videos with supplementary learning materials. Students can choose to complete several formative assessments following each mini-module or advance to the next mini-module, or any chosen point within the lesson. Following the mini-modules and formative assessments, students can choose to submit a muddiest point where they will receive timely feedback from the instructor. Students can continue the dialogue as needed directly in the LMS

The authors each followed the same construction for the Individualized Online Lessons described in this study. Learners have some autonomy over how they view and interact with the asynchronous lesson. The workflow for our Individualized Online Lesson template is displayed in Fig. 2. First, learners are directed to view an Introduction page that displays the learning objectives, links to the eBook versions of required/optional textbooks, a PowerPoint file containing the slides from the instructional videos, tips for navigating the Individualized Online Lesson, and the teacher’s contact information. Next, the learner can view several mini-modules within the Individualized Online Lesson. A mini-module contains an embedded, short instructional video (5–15 min) with supplementary resources linked or embedded in the page allowing the teacher to “chunk” information for the learner rather than providing all session content as a single entity. We provided our instructional videos using YuJa (Yuja Inc., San Jose, CA), a video content management system designed for educational institutions. The platform can be used to stream, record, edit, store, and play videos. YuJa was selected for this study for its accessibility features, including a clear audio/video feed of the teacher and their computer screen, closed captioning, transcripts, user-controlled playback speeds, and embedding into the LMS.

After each mini-module, learners have the option to complete formative assessment questions and immediately see their performance, with answer rationales. The questions ranged from assessing recall to the application of foundational concepts, using multiple-choice, matching, and short-answer formats. The learner can also decide to skip the assessment questions and progress to the next mini-module. Once the learner has finished the mini-modules, they are routed to the “Muddiest Point” prompt where they are encouraged to write about their most confusing point from the Individualized Online Lesson, following the prompt, “What is your muddiest (most confusing) point from this lesson? I will respond directly here.” The Muddiest Point was fully optional, and had no minimum or maximum word count. The Muddiest Point was created on the backend in Moodle using an “essay question” where the teacher can see the name of the student and their comment. The teacher can respond back directly through the LMS, and a copy of the teacher’s message is also sent to the learner’s email address. These questions are a private conversation between the teacher and learner, without an audience of their peers. The formative assessment questions and the “Muddiest Point” prompt in the Individualized Online Lesson allows for the teacher to provide individualized, just-in-time feedback to each individual learner. On average, students received a personal response to their Muddiest Point within 24–28 h. As seen in Fig. 2, learners can choose their own path through the mini-modules, formative assessment questions, and Muddiest Point, but at minimum are suggested to view the mini-modules. The Individualized Online Lessons were fully flexible. Although the mini-modules were designed to be consecutive and build concepts in a logical order, students could choose to complete them in any order and could re-access material and re-try activities on an unlimited basis.

### Data Collection

This study was determined to be non-human subjects research by the Oakland University Institutional Review Board (IRB-FY2022-267) where the student activities and data examined were part of the planned curriculum. At OUWB, learners have the opportunity to complete an evaluation survey of a faculty member’s teaching when the faculty member has delivered their last session in a given course or clerkship. The survey instrument has five Likert-style items and three open-ended items. This study examined responses provided for the standard open-ended questions:

1. Please comment on what the faculty member did well in the course.
2. Please comment on what the faculty member could do to improve the instruction
3. Please provide any additional comments you may have regarding the instructional materials used by this faculty member.

No questions asked the learners to reflect on our asynchronous Individualized Online Lessons specifically. Learners completed their surveys online from a location of their choosing using the OASIS (Schilling Consulting, LLC, Madison, WI) student scheduling and administration system, OUWB’s tool for giving and receiving learner and faculty evaluations. The data were collected anonymously.

Upon preliminary review of the raw data, we observed that it was common for a single narrative response from a learner to convey more than one distinct idea. During our second review, each learner’s narrative response was divided into distinct codable comments that represented a discrete idea or meaning. The comments were cut and pasted verbatim into a single Google Sheet (Google Inc., Mountain View, CA) and served as the units of analysis. In total, the data reflect the instruction delivered in three different teaching disciplines (biochemistry, cell biology, and histology), across 27 course offerings in the first year of the curriculum spanning the period between August 2020 to December 2022.

### Data Analysis

First, the comments were sorted into the binary categories of “strengths” feedback and “constructive criticism” feedback. Descriptive coding was used to summarize the essence of each comment. A code is a short word or phrase applied to a portion of qualitative data to symbolically represent its meaning [28]. After independent initial review of the dataset, the authors met to create the initial codebook for the study. A codebook is a list of all codes, their definitions, and rules for when to apply or not apply the code. Each author thoroughly reviewed their own learner comments and applied codes as appropriate in Google Sheets. Coding one’s own evaluation comments was necessary during first-round coding. Some codes pertained to the level of detail of the content taught or content taught in other parts of the curriculum, which could only be accurately applied by the faculty subject matter expert. The authors met to discuss the first round of coding, and as a result some new codes had to be created. The dataset was reviewed again and adjustments were made to comply with the updated codebook. For a second round of coding, each author reviewed another author’s coded data. Coding disagreements or points of confusion were flagged by the second coder, and the discrepancies were discussed and reconciled during research team meetings. Where appropriate, some related (but still distinct) codes were grouped into larger categories. Descriptive statistics were calculated for each code and category.

### Study Rigor

The low-stakes nature of the teaching evaluation forms contributed to the data’s authenticity. The responses were collected anonymously online, which fostered a safe and private environment where students could provide honest feedback without fear of repercussions. As the students were not prompted to write about the Individualized Online Lessons, we can infer that related comments were true thoughts that they wanted to share rather than fabricated responses to meet an evaluation requirement. Analysis of our own feedback from the students contributed to the trustworthiness of the results. Certain codes could only be accurately applied to the raw data by the faculty subject matter expert with knowledge of the curriculum and the context-specific jargon used by students. Finally, the use of second coders contributed to consistency in coding across the large dataset.

## Results

In total, 3075 learner comments, extracted from three separate academic years, were included in this analysis (Table 1). We have included sub-analysis from each of the three academic years as while there were no major differences in the overall curriculum (above and beyond the 27 select sessions analyzed here) across the 3 years, there were differences in the amount of overall remote/asynchronous session offerings across the years (with Fall 2020 having mostly remote offerings across all courses (again above and beyond these 27 sessions), vs. a more hybrid curriculum in Fall 2021 and Fall 2022. Therefore, we believe it is important to review these analyses both within individual academic years, as well as averaged across all three. The majority of comments from each year were in the “strengths” category (average per fall semester was 76.6%, SD 2.12%.) Although there was no prompt to do so, of the “strengths” comments mentioned by the learners, an average of 43.8% (SD 8.7%) were focused on the Individualized Online Lessons (Table 2). All narrative comments focused on the Individualized Online Lessons were extracted and further analyzed.

**Table 1:** Total student comments on faculty evaluations

	Total “Strengths” comments		Total “Constructive Criticism” comments		Not applicable/ no suggestions		Total Comments
	*n*	%	*n*	%	*n*	%	
M1 Fall 2020	569	75.8	121	16.1	61	8.1	751
M1 Fall 2021	908	79.0	144	12.5	97	8.4	1149
M1 Fall 2022	881	75.0	192	16.3	102	8.7	1175
*Average*	*786.0*	*76.6*	*152.3*	*15.0*	*86.7*	*8.4*	*1025.0*
*Standard deviation*	*188.4*	*2.1*	*36.2*	*2.1*	*22.4*	*0.3*	*237.6*

**Table 2:** Total “Strengths” comments related to the Individualized Online Lessons

	Total “Strengths” comments	Total Individualized Online Lesson “Strengths” comments	
	*N*	*N*	%
M1 Fall 2020	569	271	47.6
M1 Fall 2021	908	307	33.8
M1 Fall 2022	881	441	50.0
*Averages*	*786.0*	*339.7*	*43.8*
*Standard deviation*	*188.4*	*89.6*	*8.7*

Descriptive coding revealed that within the extracted “strengths” comments related to Individualized Online Lessons, there were 12 distinct codes, with an additional three sub-codes. Within the “strengths” comments, the most frequent items were learners appreciating the practice questions within the Individualized Online Lessons (average 18.6%, SD 2.3%), preference for the modular format (average 17.3%, SD 2.7%), the ability to engage with the material (average 11.1%, SD 0.9%), and finding it easy to ask questions (average 9.2%, SD 2.4%) (Table 3). Example quotes from each of the 12 distinct “strengths” codes are provided in Table S1. Representative learner comments (extracted across all three academic years, and provided *verbatim*) describing these sentiments include the following:

- “I loved that she had thinking questions interspersed between the mini-lectures!!! I have never watched a lecture like that and found it to be so helpful in making me think back to what I had been learning the past 10–15 min and synthesizing the most important concepts. I feel I already have a great grasp on the material because of this method of quizzing myself throughout the lecture. I really liked the written format as opposed to multiple choice because it made me think harder and organize my thoughts.”
- “As always, I appreciate the breaking up of the lessons with concept check question. I'll admit sometimes I groan when I see them, just cause I want to get through the material, but in the end I know they help me learn the material by forcing me to recall the things I just heard, and putting those concepts in my own words.”
- “I think she is very good at welcoming questions and securing our knowledge by implementing the modules. I felt like I could go to her for anything I felt uncomfortable with.”

**Table 3:** “Strengths” codes related to Individualized Online Lesson modality

	M1 Fall 2020		M1 Fall 2021		M1 Fall 2022		Average %	SD
	*n*	%	*n*	%	*n*	%		
Practice questions within module	52	19.2	63	20.5	71	16.1	*18.6*	*2.3*
Modular format*	49	18.1	60	19.5	63	14.3	*17.3*	*2.7*
Engaging with material	33	12.2	33	10.8	46	10.4	*11.1*	*0.9*
Easy to ask questions	28	10.3	20	6.5	48	10.9	*9.2*	*2.4*
Organization	13	4.8	30	9.8	41	9.3	*8.0*	*2.8*
Facilitates learning*	27	10.0	23	7.5	23	5.2	*7.6*	*2.4*
Quick response	24	8.9	15	4.9	32	7.3	*7.0*	*2.0*
Appreciates materials and resources (images, visuals, diagrams, videos, transcripts, captions)*	14	5.2	30	9.8	24	5.4	*6.8*	*2.6*
Evaluate learning	14	5.2	9	2.9	25	5.7	*4.6*	*1.5*
General positive impression of lesson	4	1.5	9	3.0	28	6.4	*3.6*	*2.5*
Instructor support	8	3.0	11	3.6	18	4.1	*3.6*	*0.6*
Muddiest points	5	1.9	4	1.3	22	5.0	*2.7*	*2.0*
Total Individualized Online Lesson Strength Comments	271		307		441			

^*^Strength codes with associated subcodes, further described in Table 4

Additional learner comments noted an appreciation of the organization of the Individualized Online Lessons, the Lessons’ help in facilitating learning, the ability to receive a quick response from faculty members, the materials and resources provided throughout the lessons, the ability to evaluate their learning, feeling supported by the teachers, and the opportunity to reflect on their Muddiest Points (Table 3). Representative comments (extracted across all three academic years, and provided *verbatim*) categorized into these codes include the following:

- “ I love how Dr. X organizes her lectures. I normal despise recorded lectures, and prefer to be in person. But the way that Dr. X breaks apart the lecture into mini-lectures, with concept check questions in between, and the "muddiest point" questions at end, it great. Plus, she is pretty good about giving feedback or at least responding in someway to the open-ended Concept Check Questions. This not only make me feel better about the material, but trusts that if/when I need to reach out to her about the material, she will be willing to help”
- “One thing that I can say about Dr. Y is that she gives so much attention to all her students and makes them feel like an individual and not just another student in the cohort. … Your "muddiest point" is a wonderful addition and I wish more professors would do something similar in their async. lectures. You simplify complex topics and really take the time to help students find additional resources when they ask!”

Three of the descriptive “strengths” codes could be broken down into subcodes, with important aspects noted by the students (Table 4). The modular format code included a subgroup of learners noting specifically that they appreciated that the content was broken down and/or digestible (average 57.5% of the modular format comments, SD 18.5%). Within the materials and resources code, a subgroup of learners specifically referenced the extra resources offered within the lesson and/or within follow ups to questions (average 61.1% of the materials and resources comments, SD 14.0%). Finally, within the “facilitates learning” code, learners described that they felt teachers “understood” how medical students synthesize content (average 9.9% of the facilitates learning code, SD 6.9%).

**Table 4:** “Strengths” codes with their associated subcodes

	M1 Fall 2020		M1 Fall 2021		M1 Fall 2022		Average %	SD
	* n *	%	*n*	%	*n*	%		
*Modular format*	*49*		*60*		*63*			
Modular format—broken down/digestible	26	53.1	25	41.7	49	77.8	*57.5*	*18.5*
*Appreciates materials and resources (images, visuals, diagrams, videos, transcripts, captions)*	*14*		*30*		*24*			
Appreciates materials and resources—extra resources	9	64.3	22	73.3	11	45.8	*61.1*	*14.0*
*Facilitates learning*	*27*		*23*		*23*			
Facilitates learning—understands how med students synthesize content	1	3.7	4	17.4	2	8.7	*9.9*	*6.9*

A similar analysis of the narrative data in the 12 distinct “constructive criticism” codes related to the Individualized Online Lessons was also performed (average total “constructive criticism” comments were 15.0% of total comments offered by learners, SD 2.1%, Table 1). Of the total “constructive criticism” comments, an average of 32.1% (SD 11.8) were related to Individualized Online Lessons (Table 5). Although many learners noted the appreciation of the practice question opportunities throughout the Individualized Online Lessons (Table 3), the desire for more practice questions was the most frequent comment within the “constructive criticism” codes (average 30.5%, SD 21.8%) (Table 6).

**Table 5:** Total “Constructive Criticism” comments

	Total “Constructive Criticism” comments	Total Individualized Online Lesson “Constructive Criticism” comments	
	*n*	*n*	%
M1 Fall 2020	121	26	21.5
M1 Fall 2021	144	43	29.9
M1 Fall 2022	192	86	44.8
*Averages*	*152.3*	*51.7*	*32.1*
*Standard deviation*	*36.2*	*30.9*	*11.8*

**Table 6:** “Constructive Criticism” codes related to Individualized Online Lesson modality

	M1 Fall 2020		M1 Fall 2021		M1 Fall 2022		Average %	SD
	*n*	%	*n*	%	*n*	%		
More practice questions	2	7.7	14	32.6	44	51.1	*30.5*	*21.8*
Want more multiple choice questions (want to practice exam format)	3	11.5	13	30.2	14	16.3	*19.3*	*9.7*
Takes too long to work through problems/questions	4	15.4	7	16.3	2	2.3	*11.3*	*7.8*
Would like question answers immediately available/available as separate doc/at the end	5	19.2	2	4.7	5	5.8	*9.9*	*8.1*
Navigation issues	6	23.1	0	0.0	4	4.7	*9.3*	*12.2*
Prefer one long video/don’t like format	2	7.7	1	2.3	6	7.0	*5.7*	*2.9*
More supplemental material	0	0.0	4	9.3	2	2.3	*3.9*	*4.8*
Make more interactive	1	3.9	2	4.7	1	1.2	*3.3*	*1.8*
Remove questions	2	7.7	0	0.0	0	0.0	*2.6*	*4.4*
Questions are too hard	1	3.9	0	0.0	2	2.3	*2.1*	*2.0*
Would prefer in person	0	0.0	0	0.0	5	5.8	*1.9*	*3.3*
Be more receptive to questions	0	0.0	0	0.0	1	1.2	*0.4*	*0.7*
Total Individualized Online Lesson “Constructive Criticism” comments	26		43		86			

An average 19.3% (SD 9.7%) of additional “constructive criticism” comments specifically requested more multiple choice questions with a desire to practice exam format questions. Additional comments observed some design elements which could be improved such as providing the solutions to questions in one document for quick review, and improving the navigation of the Individualized Online Lessons within the LMS (Table 6). Example quotes from each of the 12 distinct “constructive criticism” codes are provided in Table S2. Representative learner comments (extracted across all three academic years, and provided *verbatim*) for these points include the following:

- “I think Dr. Z could improve by providing more multiple choice practice questions rather than written response practice questions because we are never tested by written response.”
- “did not really like the open response Moodle questions, but it did help me formulate an answer that's not multiple choice. otherwise, no complaints”
- “I don't think the modules are an effective way to assess knowledge as we go along. I would prefer a separate ppt or pdf with all the questions for us to go through once we feel confident about the material.”

## Discussion

**Fig. 3:**
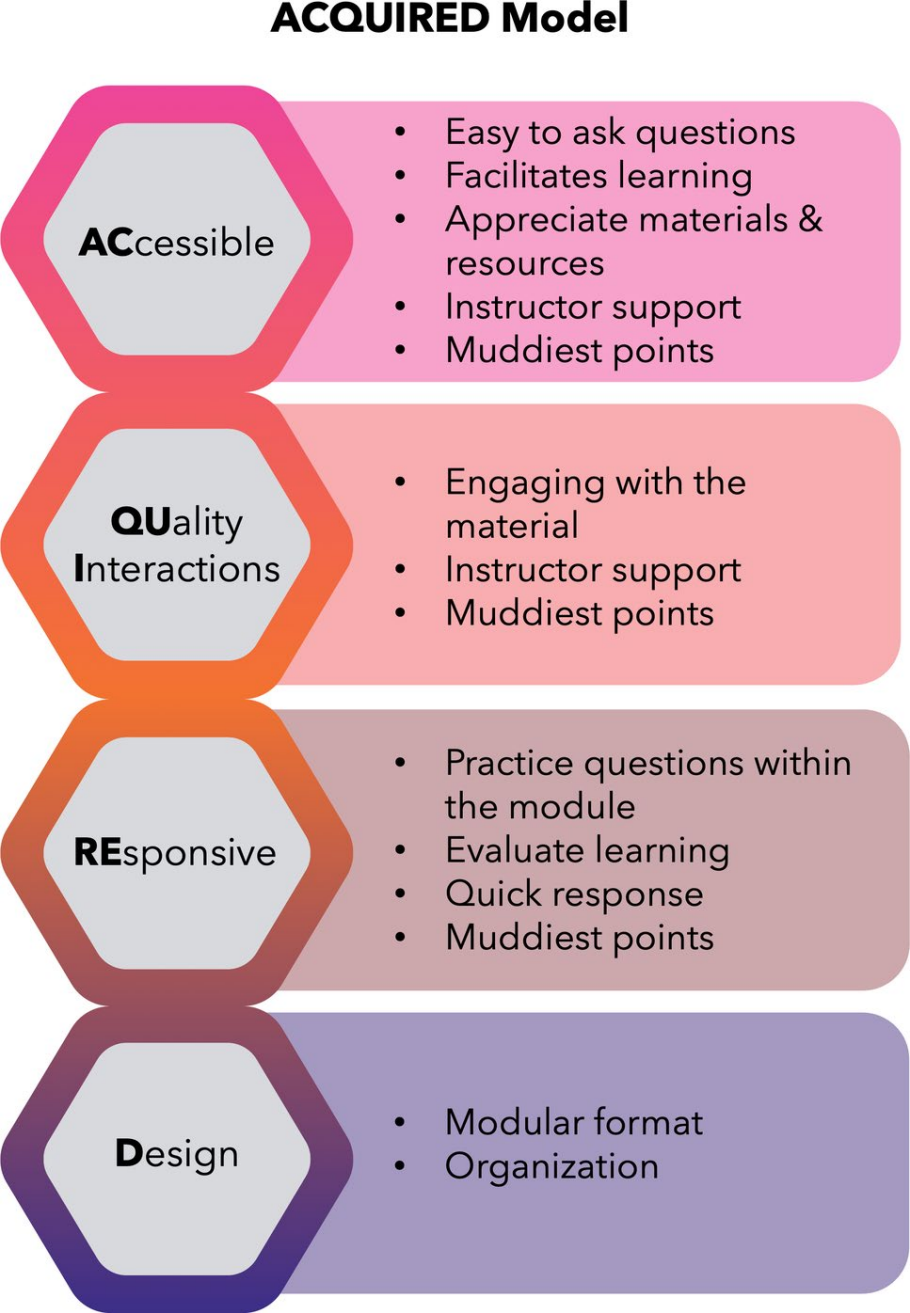
Elements of the ACQUIRED asynchronous delivery model (left) mapped to salient research codes (right)

Descriptive coding, categorization, and interpretation of both strengths and constructive criticism student evaluation comments revealed that our instructional design addresses both intrinsic and extrinsic classroom barriers (Fig. 1), provides an accessible venue for learner-teacher communication, fosters bi-directional student–teacher communication, and provides useful formative feedback through an organized and digestible format. Based on our findings, we developed a transferrable model, “ACQUIRED,” for asynchronous teaching: **AC**cessible **QU**ality **I**nteractions **RE**sponsive **D**esign. The model’s elements, mapped to the 12 strengths research codes, are presented in Fig. 3. The ACQUIRED elements do not exist in isolation, but rather are synergistic.

### ACquired—Accessible

Our students reported benefitting from the accessible and “easy” nature of learner-teacher communications and the materials. The Muddiest Point activity provided students with a one-on-one, private channel through which to convey points of confusion and ask questions. This communication mechanism may have mitigated the anxiety of otherwise having these vulnerable conversations in front of others [13]. Caton et al. argue that learners ask more questions and of a higher complexity in an online setting compared to an in-person environment due to social and technical conditions [32]. Integration of the muddiest point within the lesson also removed the burden of waiting in line after class or office hours, needing to book an appointment, or managing communication through an additional platform. Students also appreciated being able to access the variety of teaching aids (e.g., videos, transcripts, supplementary visuals) asynchronously within one place in the LMS.

### acQUIred—Quality Interaction

Our data provided evidence of Moore’s *quality dialogue* between learners and teachers [27]. The students valued teacher support, made possible by the Muddiest Point activity. This tool allowed us direct communication with each learner to understand their needs and a mechanism to respond back. Outside of teacher-learner interaction, students also valued the interactive nature of the optional formative assessment questions. The variety of question formats, such as multiple choice, matching, and short answer questions, allowed them to actively input information into the LMS. Moore also recognized the importance of *quality interactions* between learners and content, arguing that learner-teacher, learner-learner, and learner-content interaction are essential for quality distance education [33]. Meta-analyses have demonstrated that strengthening these interactions in higher education is associated with increased student achievement both in distance and in hybrid formats [34, 35].

### acquiREd—Responsive

It is well established that the timeliness of formative feedback is essential for students to be able to make adjustments to their learning accordingly [30, 36]. Our data show that learners appreciated a “quick response” offered by the teacher through the Muddiest Point, thereby supporting *responsive* teaching. Asynchronous learning has been suggested to be “less teacher-dependent” [29]. We suggest the opposite; in fact, asynchronous learning provides a rich experience where the teacher can be highly responsive to the learners without the boundaries of time and physical space. Students also described the role of receiving a personalized response in making them feel recognized as an individual. Sung and Mayer define “social presence” in online education as “the degree to which a person is perceived as a ‘real person’ in mediated communication” [37]. It is important to not overlook social presence in lieu of merely focusing on content delivery. Social presence is associated with improved learning outcomes and student satisfaction in higher education [37–39]. Perhaps a misconception is that asynchronous teaching allows the teacher to be “absent,” where a video is posted and teaching is complete. In addition, responsiveness was provided automatically through the multiple choice and matching-style formative assessment questions. Students could see their immediate performance on a question with a rationale for why their answer was incorrect, and they could choose to attempt the question again.

### acquireD—Design

Students consistently commented on the easily “digestible” presentation of content due to its organization into mini-modules. Providing material in smaller “chunks” has been shown to aid learning and organizing information, and reduce cognitive load [40, 41]. We also observed learner appreciation for instructional design elements that contribute to more inclusive classrooms, such as transcripts and closed captioning for videos, visual aids, and select use of supplemental material. In a responsive manner, we routinely reflected on the constructive comments to implement small design changes. Most often, learners requested additional practice problems or alternative question formatting. Teacher responsiveness to learner feedback is prudent to ensure increased quality of asynchronous materials and their utilization. Herein lies a key intersection between the *Design* of the lesson and teacher *Responsiveness*.

### Limitations

The ACQUIRED modality may be limited in some other contexts by intended learning outcomes, faculty preferences, and resources. Our model was developed through the backwards design process, beginning with the identification of the desired results [42]. The learning objectives for our basic science sessions were largely knowledge-based; thus, we aimed to create a modality that was suitable for didactic purposes while mitigating the intrinsic and extrinsic barriers to large-group in-person learning. It is unknown if our model would be suitable for skills-based learning outcomes. Our model was also not designed with learner-learner interactions in mind. Second, teachers may be resistant to shift from in-person teaching-centered to online student-centered approaches due to perceived limitations in learner-teacher interaction. Teachers report having more positive impressions of students that they can “see,” report a greater job satisfaction and teaching self-efficacy, and feel better connected and engaged in-person [43–45]. Last, preparing for ACQUIRED-delivered teaching requires additional time for video recording, formative assessment development, and responding to Muddiest Points. Nevertheless, as authors and designers, we found it reinvigorating to be able to interact with more students one-on-one, and found a new sense of professional identity within modern health professions education.

This study is limited by its dataset and curricular context. Without having a specific prompt on the faculty evaluations regarding the Individualized Online Lessons, the dataset may be biased towards students who had strong positive or negative perceptions and chose to write. There may be additional unrepresented learning experiences that would have been documented if a specific prompt was used. Second, it was not possible to contact learners for follow-up in-depth discussion, nor evaluate summative assessments and learning outcomes at the individual student level, due to the anonymous nature of the faculty evaluations. That being said, in aggregate, there were no major changes to learning outcomes related to these content sessions from before implementation of the ACQUIRED format to after. Finally, we studied asynchronous teaching within hybrid courses, alongside interactive, in-person application, based and skill development sessions, with delivery formats spanning fully in-person, hyflex, synchronous online, and asynchronous online. It is unknown whether the ACQUIRED format would be as positively received in a fully asynchronous course, or if the learners would experience modality fatigue.

### Future Directions

Using the existing dataset, future studies will characterize student–teacher interactions through the muddiest point correspondences. In-line with Cook et al.’s recommendations for researching internet-based learning in health professions education, an experimental study looking at student outcomes should be conducted to compare the ACQUIRED model to other asynchronous modalities, or to compare variations of the ACQUIRED format [46]. Additional studies should be conducted to determine the ideal balance for ACQUIRED format and in-person activities such that social and interpersonal skill development are maintained. Finally, as the evolution of asynchronous modalities in health professions is dependent on teacher buy-in, faculty perceptions of asynchronous learning and how it impacts their enjoyment of teaching and professional identity should be explored.

### Recommendations

While we utilized the “Lesson” feature in Moodle, other LMSs offer analogous tools, for example, “Pages and Modules” in Canvas (Instructure Inc, Salt Lake City, UT), and “Learning Modules in” Blackboard (Anthology Inc., Boca Raton, FL). The following recommendations can be applied regardless of the LMS:

- Clearly communicate with students regarding the time commitment for asynchronous learning, expectations for participation, and purpose of the formative assessments and Muddiest Point.
- Provide a tutorial for navigation.
- Break the content into mini-modules (5–15 min).
- Follow each mini-module with formative assessment and allow students to see their performance immediately.
- Embed materials/media, when possible, to keep the user interface clean.
- Provide prompt feedback to each Muddiest Point through the LMS.
- Include elements for accessibility, e.g., video closed captioning and transcripts.
- Maintain a back-up document or files with all contents in case of technical issues.
- Schedule dedicated time for responding to the Muddiest Points.

## Conclusions

In conclusion, our data informed the design of the ACQUIRED model for asynchronous instruction, emphasizing **AC**cessible **QU**ality **I**nteractions and **RE**sponsive **D**esign. This work builds on previous studies examining recent asynchronous learning during emergency remote teaching only, as well as those that found asynchronous learning to reduce the interactions of faculty with their students [25, 26]. By embracing asynchronous learning, intrinsic and extrinsic barriers to student–teacher interactions, prevalent in traditional in-person settings, are addressed. Through intentional deployment of asynchronous modalities, meaningful and bidirectional learner-teacher interactions are nurtured.

## Supplementary Information

Below is the link to the electronic supplementary material.

Supplementary file1 (DOCX 17.3 KB)

Supplementary file6 (DOCX 15.2 KB)

## Data Availability

The datasets generated during and/or analyzed during the current study are not publicly available due to the sensitive nature of the data set, but may available from the corresponding author on reasonable request.

## Declarations

This study was determined to be non-human subjects research by the Oakland University Institutional Review Board (IRB-FY2022-267).
