# Supplementary material for: ACQUIRED: An Innovative Asynchronous Modality to Increase Quality Teacher-Learner Dialogue and Overcome Classroom Barriers in Basic Science Medical Education
Source: Med Sci Educ. 2025 Feb 6;35(2):905–17. doi: 10.1007/s40670-024-02248-w (PMC12058606; doi:10.1007/s40670-024-02248-w)
Supplement: Supplementary file 1 — Supplementary file1 (DOCX 17.3 KB) [file 40670_2024_2248_MOESM1_ESM.docx]

| **Supplementary Table 1. Associated Sample Quotes for "Strengths" Codes Related to Individualized Online Lesson Modality** | | |
| --- | --- | --- |
| **Code** | **Sample Quote 1** | **Sample Quote 2** |
| Practice questions within module | The asynchronous session were easy to follow and the questions embedded into the sessions were extremely helpful in understanding the information. | I really enjoyed the questions after each video as well. They made me critically think about each concept and helped me understand them better. |
| Modular format* | I found the format of video-question- video-question to be very helpful. It solidified my understanding of the material immediately after watching the videos. It was also helpful to get her feedback on the questions. Although the answers were posted in the moodle modules it was beneficial to get her help on the questions that I got wrong. | I really appreciate Prof X's modules because they help me learn at my own pace and they're very dynamic with the concept questions in between each module. |
| Modular format subcode - Broken down/ digestible | It was clear to me that she understood how medical students digest information and provided the material in a bite-sized format, with checkpoints to help us synthesize what we just learned. I also appreciated the fact that she sent us emails after we completed the module, breaking down what we took away and what needed a bit more work. Even if the emails were standard responses, they still gave the impression that Dr. Y genuinely cares about your understanding of the material. | I really enjoyed the modules you prepared for us as I felt breaking down the concepts into smaller sections/videos made tackling the material easier and less daunting. I also really liked the practice questions you supplied to us to further solidify our understanding of the concepts. |
| Engaging with material | I greatly enjoyed Dr. Z's lectures, they were succinct while still covering relevant details. I enjoyed the breaks for summarizing and engaging with the material. | The modules were fantastic, and I really enjoyed having questions throughout that would allow me to test whether or not I A) was paying attention enough and B) understood the material. These check-ins were very helpful and I wish other professors would utilize them - it made the learning experience feel active. |
| Easy to ask questions | One thing that I can say about Dr. Z is that she gives so much attention to all her students and makes them feel like an individual and not just another student in the cohort. I appreciate her kindness and understanding, which shines through all her interactions whether its in an email or in-person. Your "muddiest point" is a wonderful addition and I wish more professors would do something similar in their async. lectures. You simplify complex topics and really take the time to help students find additional resources when they ask! | I enjoy your "muddiest points" since it gives me the opportunity to ask you a question about session material immediately after lecture! |
| Organization | I love how Dr. X organizes her lectures. I normal despise recorded lectures, and prefer to be in person. But the way that Dr. X breaks apart the lecture into mini-lectures, with concept check questions in between, and the "muddiest point" questions at end, it great. Plus, she is pretty good about giving feedback or at least responding in someway to the open-ended Concept Check Questions. This not only make me feel better about the material, but trusts that if/when I need to reach out to her about the material, she will be willing to help | I thought Dr. Y's asynchronous lectures were well organized with lots of opportunities to ask questions and check comprehension. Her expectations for students were clear. |
| Facilitates Learning* | I think Dr. Z was very organized in the lecture, which made it seem much simpler to understand in sections compared to trying to digest a lecture as a whole. By breaking it up into manageable chunks made the lectures much more easier to understand and learn from for me. | Dr. Y is a FANTASTIC lecturer. She is one of few in the course that understands how to present material to students in lecture format that makes sense. She is engaging in her teaching style even in an online format which makes her lectures very easy to digest. She understands how students learn most effectively and incorporates that into her teaching style. Her and Dr. X need to host a seminar on how to most effectively present material in lectures for the other professors to model their lectures after. |
| Facilitates learning subcode - Understands how med students synthesize content | Dr X really understands how to get information into someone’s brain and keep it there. I think she is a good model of what every medical professor should strive for in lectures. | Dr. Z took some of the most difficult concepts, pathways, and reactions we had to learn and presented them in a way that made them less intimidating and less scary. This is huge because the material she had to present can easily overwhelm students as it is highly technical and many people have not been exposed to this kind of material before. She took her time in explaining things, she simplified her language when necessary, and you could tell she really tried to make the students feel at ease, because again, this stuff can be pretty anxiety inducing. Her presentations were organized and she broke them into manageable pieces. This helped me keep things organized in my mind and made tackling a new lecture less difficult. I always walked away from the material knowing what I was expected to learn. |
| Quick response | As I stated in the previous question, Dr. Z's modules proved to be engaging and effective in solidifying my understanding of the material she presented, ranging from basic material, to more complex material. Additionally, Dr. Z was always very responsive to our questions and always advised going to her office hours if we had any questions. I appreciated these aspects of her teaching as well. | I appreciated how Dr. Y divided up her material and made it very easy to learn from. She responds quickly to emails and pays attention to student input from the "muddiest point" submissions. I would be excited to watch her lectures as I knew my time would not be wasted. |
| Appreciates materials & resources (images, visuals, diagrams, videos, transcripts, captions)* | Similar to Dr. Y, Dr. Z's use of instructional material is exceptional. I think every professor should model their online lectures after her. I like that you can have the captions on the videos when you use moodle. That way you have auditory, visual, and reading styles of learning. She also does a great job of following up on answer responses. And the balance of open-ended and applied concept questions is perfect. Makes you recall the entire lecture and then apply it. | Overall, this professor presented the material in a clear and concise manner. She was very enthusiastic about teaching. Additionally, her modules were extremely helpful. The diagrams and images she used in her lecture materials were very informative and helpful. |
| Appreciates materials & resources subcode - Extra Resources | I really like the way she presents the material and makes extra videos for us to digest concepts. I also like how she sprinkles in practice questions between lecture content. Even though she was virtual, she is extremely responsive to emails. | Dr. X did a good job of explaining concepts and using outside resources, such as videos, to better explain some points. I also think having the pictures to show the process helped me learn the material, rather than only using words. The module format was also nice to put any concepts that I was struggling on for Dr. X's review. |
| Evaluate learning | I appreciated that many of Dr. Z's lectures/modules were divided into parts with questions in between. It was nice to be able to check comprehension through these questions before moving on to the next part. In addition, I also liked that the lecture transcripts were included on Moodle as I used these occasionally. Overall, Dr. Z's lectures were clear and allowed for a good learning experience. | She did very well with her modules that allowed me to test what I retained in real time. It was very helpful to also use her pathway integration tool. |
| General positive impression of Lesson | I loved the module based lectures with the async and the clinical and real world questions and topics that were asked, especially the concept check Q's. Also the funny slides were a nice touch for engagement during the lecture. | The modules are a really effective way to teach. |
| Instructor Support | I appreciate Dr. Y's slides that are super well organized and easy to read. It also speaks volumes that she individually responds to students' moodle questions. It shows that she really cares for her students and their success. I also appreciated the practice questions/powerpoint | It is evident that Dr. X really cares about our learning and that we understand the material. She is very approachable and always makes sure we feel comfortable coming to her with questions. She is also very helpful during office hours in explaining concepts that are difficult, while also encouraging us to try explaining them in our own words. |
| Muddiest Points | Dr. Z does an excellent job carrying us through her lectures with carefully designed modules. I LOVE the questions that she writes in and that she gives us space to try and answer. It is also amazing that she creates a space for us to address our muddiest points and that she responds in kind! It is so effective and encouraging to have this from Dr. Z. I feel more sure about my capacity to perform well on her sections of the material. | One thing that I can say about Dr. Y is that she gives so much attention to all her students and makes them feel like an individual and not just another student in the cohort. I appreciate her kindness and understanding, which shines through all her interactions whether its in an email or in-person. Your "muddiest point" is a wonderful addition and I wish more professors would do something similar in their async. lectures. You simplify complex topics and really take the time to help students find additional resources when they ask! |
| * Parent code has associated subcode | | |
