# Supplementary material for: ACQUIRED: An Innovative Asynchronous Modality to Increase Quality Teacher-Learner Dialogue and Overcome Classroom Barriers in Basic Science Medical Education
Source: Med Sci Educ. 2025 Feb 6;35(2):905–17. doi: 10.1007/s40670-024-02248-w (PMC12058606; doi:10.1007/s40670-024-02248-w)
Supplement: Supplementary file 2 — Supplementary file6 (DOCX 15.2 KB) [file 40670_2024_2248_MOESM2_ESM.docx]

| **Supplementary Table 2. Associated Sample Quotes for "Constructive Criticism" Codes Related to Individualized Online Lesson Modality** | | |
| --- | --- | --- |
| **Code** | **Sample Quote 1** | **Sample Quote 2** |
| More Practice Questions | Overall, I don't think Dr. X's instruction needs improvement. If anything, I would ask for additional practice or concept check questions to go through at the end of the lecture. | My sole suggestion for Dr. Z would be adding in more questions in this lecture throughout so that students can challenge themselves. The several questions she does have do a great job of synthesizing content and were a tool I found myself frequently going back to. |
| Want more multiple choice questions (want to practice exam format) | While I appreciate free response questions, which allow me to summarize and access my understanding, I would benefit from having more multiple Concept Check Question's. Since our assessment is in Multiple Choice format, having questions in this format is just another chance to practice test taking strategies. Either way, her practice questions were great in actively recalling each sections of her lectures. | More multiple choice questions to simulate exam questions. Open ended ones in the modules were good, but supplemented with multiple choice would have helped. |
| Takes too long to work through problems/questions | Although they ended up taking much longer than the allotted 50 minutes due to all the questions throughout and transitions between the different parts for one lecture, I appreciated all the extra things provided! It would be nice for them to be slightly shorter if possible and maybe more multiple choice questions rather than written out questions. I spent a long time typing out answers that was useful, but could have been just as useful to have multiple choice questions to check understanding (or fill in the blank?). | I wish it was more clearly communicated that her asynchronous content would take much longer, potentially by taking a longer time slot in the calendar. I would spend doubly as long as the scheduled lecture to get through everything, which is fine but would have been nice to know in advance. |
| Would like question answers immediately available/available as separate doc/at the end | It would be helpful to have a document attached with all practice questions performed in the module. Then students who like to attempt practice questions before the exam have a resource that is easy to find! | Not the biggest fan of the module format, would suggest having a pdf of the questions rather than dispersed through the module. |
| Navigation issues | I'm not a huge fan of the moodle modules. I just find them hard to navigate, and if I get a question wrong, I'm forced to try again until I get it right. This is frustrating on some of the matching questions with seven or eight components. | The moodle multiple page set-up can be hard to navigate, but maybe it's just me. At first, it seemed like if you wanted to go back and check one specific thing you had to click through the entire lesson and answer certain questions to get to what you were looking for, but for some lessons I'm given the option to skip around. This is not Dr. X's fault, but maybe a quick explanation on how to navigate her material would be helpful |
| Prefer one long video/don't like format | I would rather the lectures be condensed into one video than multiple. I definitely do like the questions interspersed but I think it might be helpful to combine the videos all into one so it's more continuous then have the questions clumped together at the end. | I personally do not like when lectures are broken into different videos. I'm sure there are things that I am not aware of that make it a better way to do it, I just thought that I would mention it. |
| More supplemental material | I think Dr. Z should continue to add videos at the end of her modules that help visualize the content better. She did this for the session 20 video and it was very helpful. She asked us to watch the video first without content, before giving the lecture, and then asked us to watch it after learning the content to put together everything. This was a really effective method of teaching content because it gives a general overview of what to expect without intimidating students with names, steps, etc. Dr. Z would benefit from finding more of these videos and continuing to use this method in her other lectures as well. | I would enjoy more handouts to accompany her lectures. |
| Make more interactive | Dr. Y could improve by making the lectures more interactive. | I feel the instructor could add more think-pair-shares to her lecture to keep students engaged and to reinforce concepts. I enjoy doing a concept check after receiving information to see if I have an information gaps in my knowledge. |
| Remove questions | Please get rid of the in between section questions. Those are very annoying when we are reviewing. | Please get rid of the in between questions. |
| Questions are too hard | I believe that the instructor did a great job overall, but one site of improvement could be that I felt like some of the reflection questions between the lecture videos were not directly answered in the lectures. It made it a bit hard to verify answers and understand where the information was coming from. | Occasionally the questions written seemed more complex than they needed to be for how challenging the concept was in the question- some questions seemed too wordy, etc. |
| Would prefer in person | It would be great to have the option to attend an in person lecture with Dr. Y, but I appreciate her flexibility with providing students with virtual lectures. | It would have been nice to have an in-person lecture option. |
| Be more receptive to questions | Maybe be more receptive to questions? I sent a few questions in the muddiest points early on and never got actual answers back. | *(only one comment in this code)* |
